# Astaxanthin as a new Raman probe for biosensing of specific subcellular lipidic structures: can we detect lipids in cells under resonance conditions?

**DOI:** 10.1007/s00018-020-03718-1

**Published:** 2020-12-08

**Authors:** Krzysztof Czamara, Adriana Adamczyk, Marta Stojak, Basseem Radwan, Malgorzata Baranska

**Affiliations:** 1 Jagiellonian Centre for Experimental Therapeutics (JCET), Jagiellonian University, 14 Bobrzynskiego Str., 30- 348 Krakow, Poland; 2 Faculty of Chemistry, Jagiellonian University, 2 Gronostajowa Str., 30-387 Krakow, Poland

**Keywords:** Carotenoids, Endothelium, Raman spectroscopy, TNF-α, Inflammation, Lipids, Raman imaging, Fluorescence imaging

## Abstract

Here we report a new Raman probe for cellular studies on lipids detection and distribution. It is (3S, 3'S)-astaxanthin (AXT), a natural xanthophyll of hydrophobic properties and high solubility in lipids. It contains a chromophore group, a long polyene chain of eleven conjugated C=C bonds including two in the terminal rings, absorbing light in the visible range that coincides with the excitation of lasers commonly used in Raman spectroscopy for studying of biological samples. Depending on the laser, resonance (excitation in the visible range) or pre-resonance (the near infrared range) Raman spectrum of astaxanthin is dominated by bands at ca. 1008, 1158, and 1520 cm^−1^ that now can be also a marker of lipids distribution in the cells. We showed that AXT accumulates in lipidic structures of endothelial cells in time-dependent manner that provides possibility to visualize e.g. endoplasmic reticulum, as well as nuclear envelope. As a non-toxic reporter, it has a potential in the future studies on e.g. nucleus membranes damage in live cells in a very short measuring time.

## Introduction

Visualization of subcellular compartments gives valuable insight into cellular processes, which is important for detection and tracking the changes that occur during various cellular events such as cell signalling, metabolism as well as pathology development, consequently, enhancing diagnosis and treatment [1, 2]. Studies on subcellular lipidic structures such as lipid droplets (LDs), endoplasmic reticulum (ER) and nuclear envelope have gained substantial importance due to their roles in many aspects related to health and diseases development [3, 4].

Lipids are crucial for maintaining proper cell functioning by participating in cellular signalling, building membranes, and being an energy reservoir. Abnormalities of lipids content, distribution and structure can be a marker of pathological processes undergoing in the cells, e.g. inflammation or development of various lifestyle diseases [5–7]. The idea to use a different type of conjugated probes, e.g. fluorophores (rhodamine labelled phosphatidylethanolamine), hydrophobic dyes like BODIPY (boron dipyrromethene), Nile red or Oil red O (ORO), is to visualise lipids distribution in cells or tissue. Another popular approach is to use antibodies, which requires cells fixation and permeabilization through a membrane [8, 9]. However, the procedure is demanding and can lead to the sample contamination and misinterpretation of the results. Lipids polarity requires that probes for their detection need to be lipophilic. Plenty of these probes are not metabolically inert, this leads to undesirable influences on cellular processes or viability and subsequently may change the outcome of the experiment. The limitations of the widely used fluorescence microscopy and other techniques trigger the development of new probes and techniques to determine lipids redistribution and accumulation [9].

On the other hand, Raman spectroscopy is a method of choice to detect lipids in biological samples due to long polyene chains resulting in large Raman scattering cross section. Lipids can be detected based on their characteristic Raman profile. Their marker bands are omnipresent in the Raman spectra of cells and tissues and can be used as label-free markers of developing pathology [10–12]. In some cases, new groups of lipids are formed upon the stimuli of inflammation, e.g. after TNF-α treatment of endothelium [3]. Characteristic Raman bands for lipids [7] are observed in the following spectral regions: 1500–1400 cm^−1^, 1300–1250 cm^−1^, 1200–1050 cm^−1^ and 3000–2800 cm^−1^. Raman imaging enables for label-free visualization of organelles and cellular compartments, e.g. LDs, ER, nuclei, and for monitoring of the distribution of various substances at the subcellular level [3, 4, 13–15]. However, some experiments require special conditions of measurements, for example fragile tissues need to be examined under the low laser power to reduce the changes of local overheating and damage of the sample or when live cells are studied instead of fixed ones. Moreover, for some lipidic subcellular structures the diffraction limit is too low to image them directly, e.g. nuclear envelope or the lipid membrane. Therefore, using Raman probes that show intense bands or well isolated from the other cellular components, to selectively image subcellular structures, has recently gained much attention.

Carotenoids are a group of compounds widespread among plants, algae, many microbes as well as in animals, which structurally are tetraterpenoids build of eight isoprenoids units. Their conjugation of carbon–carbon double bonds is reflected in the wide range of colours from yellow to red [16, 17]. The presence of oxygen atoms allows for the classification of carotenoids into carotenes and xanthophylls; the first group are non-oxygenated compounds as e.g. β-carotene or lycopene, while the xanthophylls are oxygenated one, e.g. astaxanthin [16–18]. Due to important properties of carotenoids functions in the living organisms, especially in animals and humans, they have been widely studied.

Astaxanthin (AXT) is a red pigment well known for its potent antioxidant effect. Many studies suggest not only antioxidative but also anti-inflammatory properties, the ability to suppress cancer, and anti-diabetic activity of this compound in vitro [18–20]. AXT can be easily observed in Raman spectroscopy due to enhancement, i.e. it absorbs light in the visible range that coincides with the excitation of lasers commonly used in Raman spectroscopy for studying of biological samples [16, 21]. Depending on the laser, resonance (excitation in the visible range) or pre-resonance (the NIR range) Raman spectrum of astaxanthin [22] is dominated by bands at ca. 1008, 1158, and 1520 cm^−1^. It has been shown that carotenoids have a high affinity towards lipids, which are commonly accumulated in the human blood and tissues [21]. Unfortunately, carotenoids often require low laser power to be observed because of bleaching and the possibility to burn down.

This work shows the potential of (3S, 3'S)-astaxanthin to be used as a new Raman probe for the detection of lipids in cells, especially in organelles containing small amounts of these compounds. Distribution of AXT studied in the human microvascular endothelial cell line (HMEC-1) indicates its accumulation in specific lipidic structures, i.e. LDs and ER, and time dependence of this process. In particular, AXT was proved to be a unique probe for the nuclear lipid membrane, which is not visible by non-labelled Raman imaging. To our best knowledge, this is the first report when the nuclear envelope was visualised.

## Materials and methods

### Sample preparation

To study the cellular accumulation of AXT, the human dermal endothelial cells (HMEC-1 ATCC^®^ CRL-3243^™^, USA) were chosen. The cells for Raman measurements were prepared as previously [3]. In brief, HMEC-1 directly seeded onto CaF_2_ slides were cultured in complete MCDB131 medium (Gibco Life Technologies) supplemented with 10 mM L-glutamine (Gibco Life Technologies), 1 μg·mL^−1^ hydrocortisone (Sigma Aldrich), 10 mg·mL^−1^ epidermal growth factor (EGF, Sigma Aldrich), 10% fetal bovine serum (FBS, Gibco Life Technologies) and antibiotic antimycotic solution (AAS with 10.000 U penicillin, 10 mg streptomycin and 25 μg amphotericin B per mL) and were maintained in an air cell culture incubator. After 24 h incubation, when the confluence at the level of about 70% was obtained, cells were divided into two groups. One group was exposed to 10 ng mL^−1^ human tumour necrosis factor alpha (TNF-α, Sigma) for 24 h and the second one was maintained in fresh MCDB131 medium. Subsequently, cells were treated with (3S, 3'S)-astaxanthin (Sigma) dissolved in DMSO and then suspended in medium at a concentration of 10 µM for 0.5, 1, 3 and 6 h. As a control group, unstimulated HMEC-1 maintained in medium was used. After stimulation, cells were fixed with 2.5% glutaraldehyde for 4 min and stored in PBS buffer until data acquisition. Beside fixed samples, also live cells were analysed: after a treatment with TNF-α (24 h) followed by an incubation with AXT (6 h) cells were transferred to fresh warm PBS and subjected to Raman measurement.

### Raman measurements

All images were acquired with the WITec alpha 300 Confocal Raman Imaging system (WITec GmbH, Ulm, Germany) equipped with the UHTS 300 spectrograph (600 lines per mm grating) and a CCD detector (Andor, DU401A-BV-352). The air-cooled solid state laser with the excitation wavelength of 532 nm was used to excite the sample. Spectra were acquired by the 63 × water immersion objective (Zeiss, NA = 1.0) with a 0.5 and 1.0 μm sampling density in xy and xz planes, respectively, 0.5 s integration time and the laser power at the sample of ca. 3 mW (low laser power) and ca. 30 mW (high laser power).

### Data analysis and processing

Data processing was performed using the WITec Project Plus software. All spectra were baseline corrected using autopolynomial of degree 2 and processed by a routine cosmic rays removal procedure. Raman images were obtained by calculation of integral intensity of Raman bands in spectral ranges 1545–1495, 2900–3030 and 3030–2830 cm^−1^ for AXT, lipids and organic matter, respectively. To study time dependence of AXT accumulation, the intensity of the AXT signal was normalized for all cells starting from zero value for control to maximum of 6 h incubation time.

### Overexpression of ICAM-1

The HMEC-1 were seeded onto 96-well plates at a concentration of 3·10^4^ cells per well, in 200 µL of medium per well and left for 24 h to grow to the 100% confluence, and then were treated as follows: AXT (10 µM, 3 h), TNF-α (10 ng mL^−1^, 24 h), AXT with TNF-α pretreatment and AXT followed by TNF-α. Cells in fresh MCDB131 medium and DMSO were taken as a negative controls. Then fixed HMEC-1 (2.5% glutaraldehyde, 4 min) were incubated in the dark with mouse anti-human CD54-PE (BD Pharmingen) and stained with Hoechst 33342 (Life Technologies) for 30 min. The overexpression of surface ICAM-1 molecule and cell nuclei were observed by fluorescence microscopy (a ScanR screening system) in randomly selected six visual fields for each well. The images were analysed using Columbus 2.4.2 Software (Perkin Elmer). The results of ICAM-1 expression were presented as mean fluorescence intensity per cell for each analysed group.

## Results and discussion

### Accumulation of AXT in various cellular organelles

**Fig. 1:**
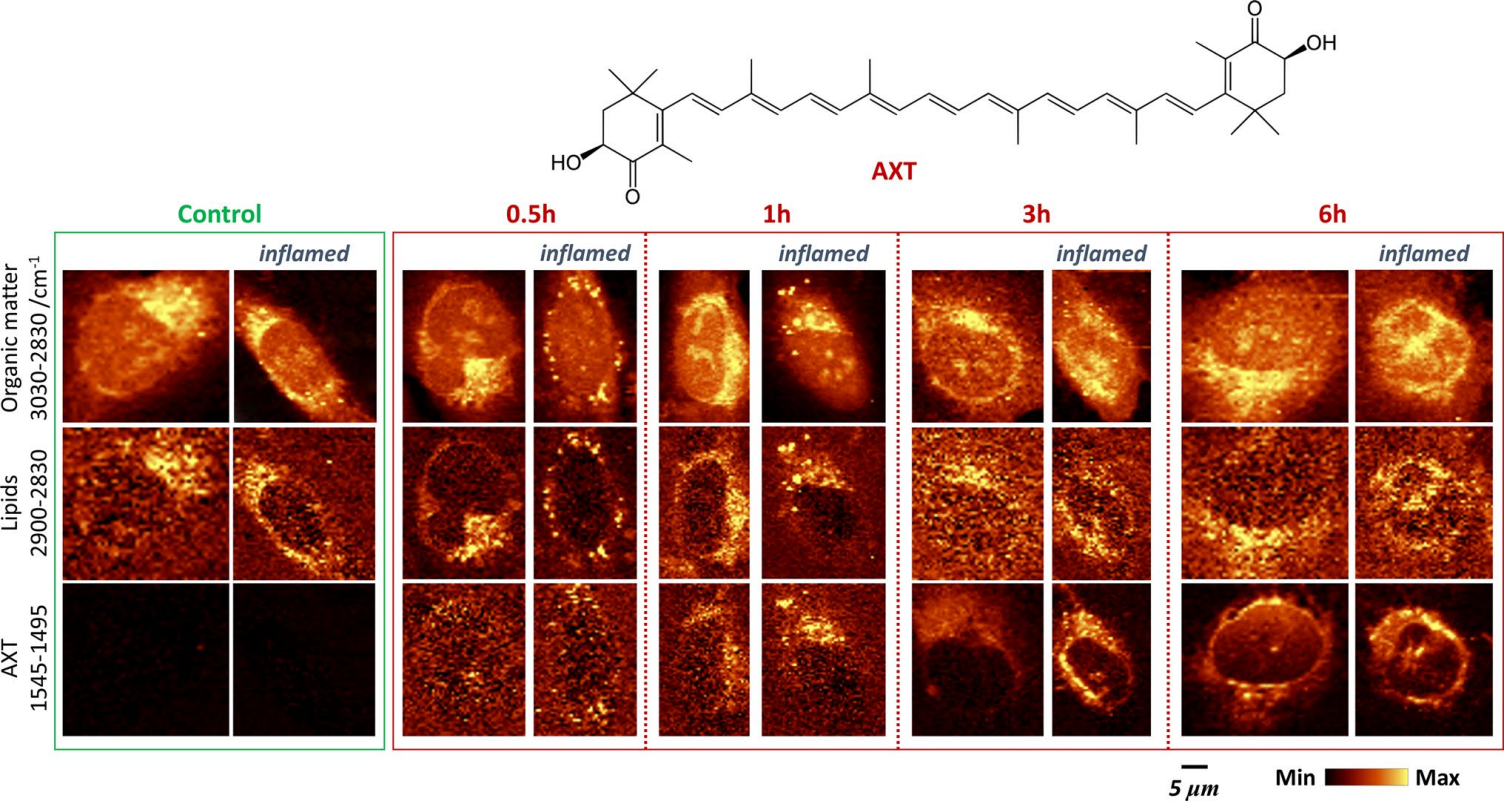
Time dependence of AXT accumulation inside cells. Raman images of all organic matter, lipids and AXT obtained from control and inflamed HMEC-1 after the 0.5, 1, 3 and 6 h incubation with AXT. Yellow color denotes high concentration of studied compound

In Fig. 1, the chemical structure of AXT and Raman images of HMEC-1 incubated with this carotenoid in various conditions are presented. Beside normal conditions, cells were pretreated with TNF-α to model their inflammation.

Raman images show the distribution of organic matter by an integration of C–H stretching bands in the 3030–2830 cm^−1^ spectral range and the lipid-rich areas based on the integration of the narrow high wavenumber region (2900–2830 cm^−1^). For AXT, due to the pre-resonance effect upon the 532 nm laser excitation, a strong enhancement of carotenoids signals was observed enabling its distribution using the most intense AXT band at ca. 1520 cm^−1^. The AXT was detected in cells even after their exposure to this compound only for 30 min, when the cells were first pretreated with TNF-α. It is seen that AXT easily penetrates into cells and locates itself in lipid droplets. After 1 h incubation of cells with AXT its presence can be detected in endoplasmic reticulum, interconnected network of membranes composed mainly of phospholipids. Raman images of AXT and lipids distribution are in good agreement, they are almost overlapping, but the situation changes for longer incubation times (3 and 6 h). For the latter, the images created based on lipids bands show organelles rich in lipids quite clearly, however the one showing distribution of AXT and hence lipids, are more detailed. Using AXT as a lipid probe, it was possible to visualize the nuclear envelope that was not possible in the first case due to low content of lipids there and hence low intensity of lipid signal.

**Fig. 2:**
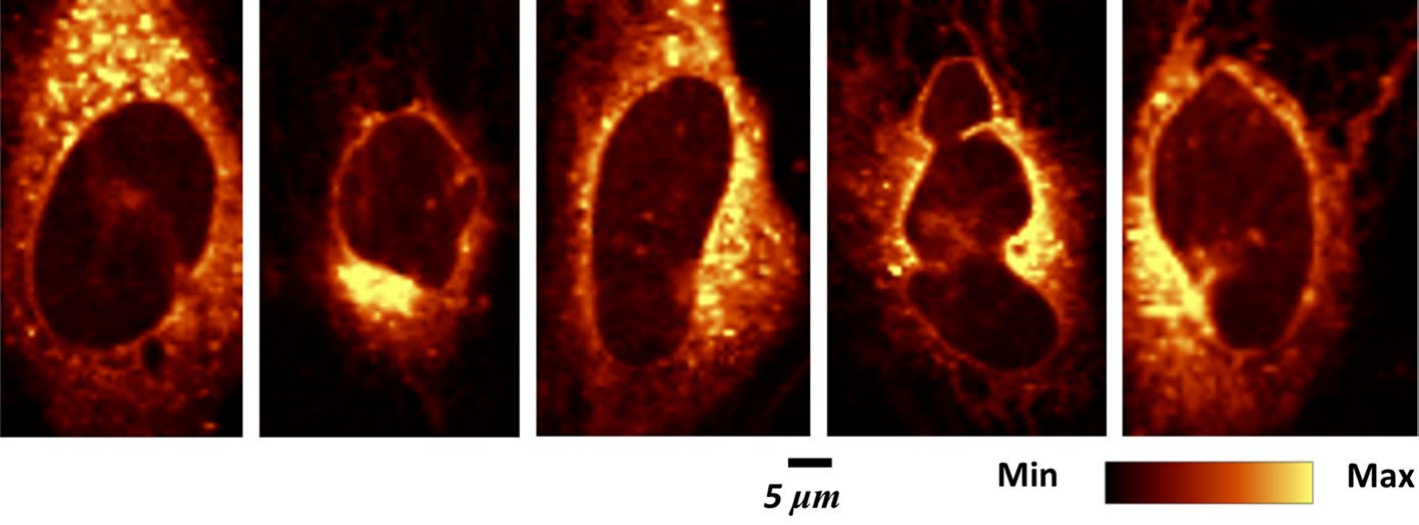
Raman images of live HMEC-1 preincubated with TNF-α (24 h) and exposed to AXT (6 h) obtained by an integration of the band in 1545–1495 cm^−1^

**Fig. 3:**
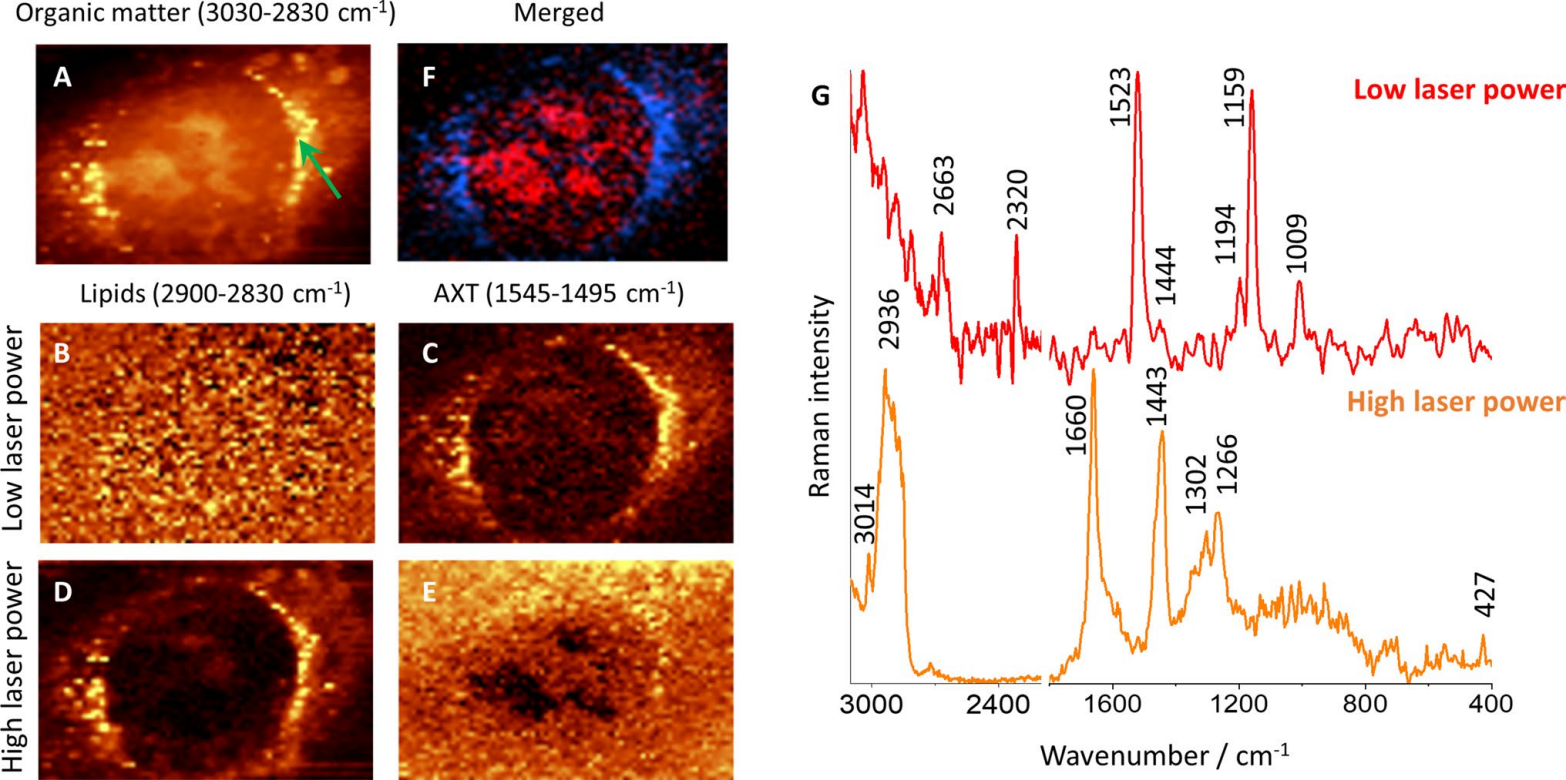
Visualization of lipids in HMEC-1 incubated with AXT. Raman image of all organic matter (**a**) obtained by integration of bands in 3030–2830 cm^−1^ spectral range and Raman images of lipids (**b**, **d**) and AXT (**c**, **e**) measured with low (~ 3 mW) and high (~ 30 mW) laser power, respectively. The merge image (**f**) of distribution of DNA (red color, 775–800 cm^−1^) and AXT (blue color, 1545–1495 cm^−1^) of the same endothelial cell measured with low and high laser power, respectively. Single Raman spectra (**g**) of lipid droplet obtained with low and high laser power

The images in the Fig. 1 demonstrating AXT accumulated in the lipidic structures including the nuclear membrane were obtained from the fixed cells. The contrast of these images is very good, and even better than the one obtained by an integration the marker band of lipids (CH_2_ stretching vibration). Moreover, the quality of the image showing colocalization of AXT and lipids for live (Fig. 2) and fixed cells (Figs. 1, 3) are very similar that proves the ability of AXT as a new lipid probe. There could be more benefits from working with living cells when the processes that involve any changes in lipids are studied, e.g. monitoring of cellular processes, formation of lipid droplets, changes within the endoplasmic reticulum, or studies on the integrity of the nuclear membrane and nucleus related to an apoptosis.

### Colocalization of AXT and lipids

When the measurement of cells incubated with AXT was performed using a low laser power (~ 3 mW), it was possible to detect AXT intracellularly, but at the same time, other biomolecules were not visible since the Raman spectra were dominated by AXT signals (data not shown). To prove that AXT really colocalizes with lipids, the same HMEC-1 was measured twice with low and high laser power (Fig. 3).

As described above, for cells incubated with AXT for 3 h, the Raman distribution images of organic matter, lipids and AXT were obtained by an integration of individual marker bands. Raman images show that both applied approaches (with low and high laser power) are complementary. By applying low laser power, the AXT distribution (Fig. 3c) is clearly visible and overlaps with lipid-reach area reflected later in the measurements with high laser power (Fig. 3d). For the latter, Raman image of AXT distribution is blank (Fig. 3e) due to its bleaching. The advantage of this approach is the possibility to overlay of images form two measurements of the same cell (Fig. 3f) showing distribution of AXT (blue area, low laser power, 1545–1495 cm^−1^) and DNA (red area, high laser power, 775–800 cm^−1^) indicates that the nucleus is encircled with AXT similarly to the endothelial cells with co-stained lamina A/C and nucleus under fluorescence microscopy [23]. The Raman spectra extracted from the same point, using low and high laser power, show characteristic but different spectral profiles. In case of the low laser power, Raman spectrum exhibits typical bands of carotenoids i.e. 1009, 1159 and 1523 cm^−1^, and even some overtones are present. In turn, Raman spectra collected for high laser power show characteristic bands of lipids with a very intense ones at 3014, 1660 and 1266 cm^−1^ indicating their high level of unsaturation. This finding undoubtedly confirms the colocalization of AXT and lipids, and applicability of AXT as a probe for lipid-rich subcellular compartments.

### Astaxanthin enables visualizing the nuclear envelope

**Fig. 4:**
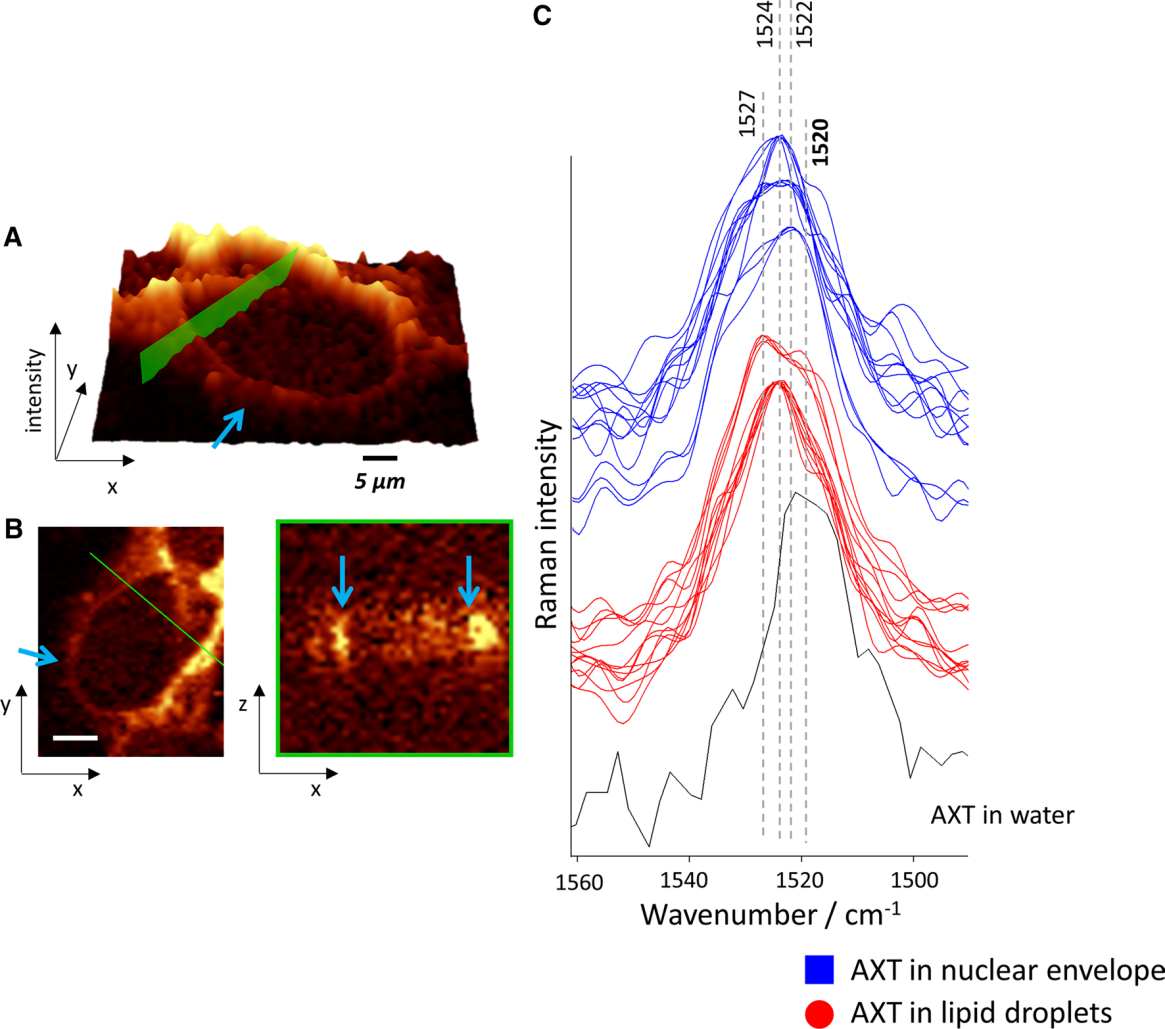
3D imaging of HMEC-1 incubated with AXT. Raman images of HMEC-1 stimulated with TNF-α (24 h) and then treated with AXT (3 h) were obtained confocally in the xy plane (intensity projection, **a**) and for selected line (marked by green color) from layers every 1 μm step in the *z*-direction (**b**) by an integration of the spectral range 1545–1495 cm^−1^ (distribution of AXT). Arrows point to the AXT accumulated in the nuclear envelope. The insert is showing the most intense AXT band of the spectra extracted from randomly selected spots of LDs and from nuclear membrane compared to the AXT spectrum in water (**c**)

Typically, the nuclear envelope is imaged by immunostaining of lamins type A (A and C), type B (B1/B2) or emerin, and was done form various type of cells including endothelial cells [24]. This staining is often performed with Hoechst nucleus staining to compare the nucleus and nuclear membrane distributions [23]. To confirm that AXT enables for visualization of the nuclear envelope of the cells, a 3D depth profiling of HMEC-1 with AXT was performed. 3D imaging provides detailed information about the distribution of cellular components, not only in one specific layer but through the whole cell. As shown in Fig. 4, AXT accumulates indeed in the outer part of nucleus which can be assigned to the nuclear envelope (blue arrows) but also reflects other lipid-rich intracellular structures. The intensity projection of Raman image (Fig. 4a) shows that AXT signal from nuclear envelope is not so intense as for LDs; however, the intensity is enough to visualize the borders of nucleus in the image of good quality contrast. The Raman image of AXT distribution in *xz* plane (Fig. 4b) clearly indicates the location of nuclear envelope even reproducing its typical curvature (left blue arrow). So, this relatively simple and fast probe for visualization of nuclear envelope could be applied to study on membranes disruption. However, it should be noted that AXT-based lipid visualization is burdened with the difficulties in separating the AXT Raman signal originating from AXT that accumulates in the nuclear envelope and other organelles, especially in the endoplasmic reticulum and lipid droplets, so visualization only the nuclear envelope with AXT is not possible.

Carotenoids tend to aggregate or change their conformation due to different environment [25]. Such situation is manifested by a shift in position of the most intense marker band due to C=C stretching and can be studied with Raman spectroscopy. The analysis of AXT spectra extracted from the LDs and from nuclear envelope was performed (Fig. 3c). As previously studied [25], AXT in DMSO occurs in monomeric form, but in the mixed solvent, e.g. water and DMSO, carotenoid molecules are arranged in supramolecular chiral structures. Here, AXT accumulates in the hydrophobic environment, such as lipids, that may have influenced AXT structure. The spectrum of AXT in water (initially suspended in DMSO) shows the intense band at 1520 cm^−1^, which is upshifted in the AXT spectrum collected from cellular structures. The spectral profile of nuclear envelope is broader with shoulders and maximum at ca. 1522 cm^−1^ in contrast to AXT spectra of LDs manifested by a band with maximum at 1524 cm^−1^ that may indicates on different AXT conformation in both cellular organelles; however, it requires more investigation including spectra modelling.

### AXT as an anti-inflammatory agent

**Fig. 5:**
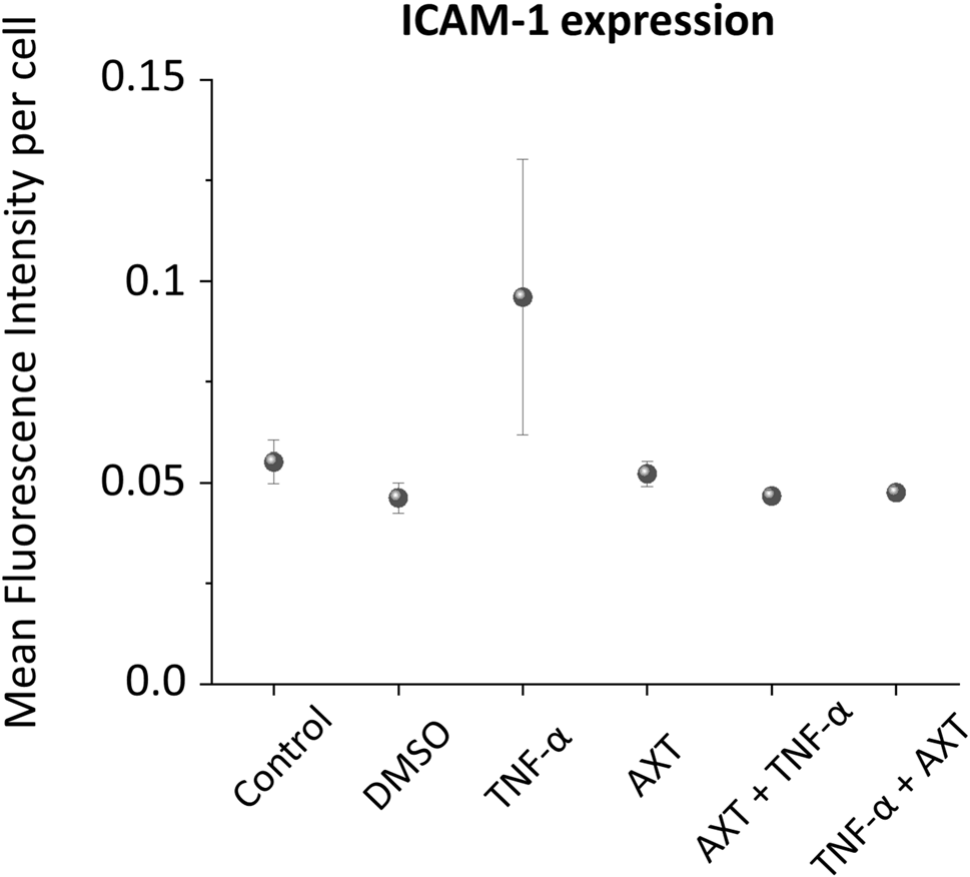
ICAM-1 expression of HMEC-1 cells treated with AXT. The staining of surface ICAM-1 molecules was performed for control in DMSO (the solvent of AXT), treated with TNF-α for 24 h, with astaxanthin (AXT) for 2 h, with AXT (3 h) then TNF-α (24 h) and with TNF-α (24 h) then AXT (3 h). The overexpression of ICAM-1 were analyzed based on the mean fluorescence intensity of the fluorophore (R-phycoerythrin) fused to the ICAM-1 antibody per cell. Values given as mean ± SEM (whiskers)

To assess the effect of AXT on HMEC-1, the marker of inflammation, i.e. intracellular adhesion molecule-1 (ICAM-1) expression was measured using fluorescence microscopy. The results are presented (Fig. 5).

Cells treated with TNF-α showed the highest expression of ICAM-1 that indicates their inflammation. This effect was significantly decreased when cells were treated with AXT that demonstrates the anti-inflammatory effect of this carotenoid on endothelial cells. The group of cells treated with AXT after the TNF-α pretreatment showed the lowest ICAM-1 expression. There was no significant difference in the ICAM-1 expression between cells treated with AXT before or after TNF-α treatment. However, in both cases, the anti-inflammatory effect of AXT is clearly visible.

These results show that AXT does not cause an inflammation to cells; therefore, it can be considered as a new Raman biosensor of lipids.

## Conclusions

Nowadays, a lot of effort is put to develop and synthesize new biosensors enabling detection and visualization of specific subcellular structures. It is expected that new probes should have many properties, including *inter alia* being non-toxic and non-cytotoxic, easily entering cells, being inert and not affecting cellular processes, cheap and easy to manufacture, and most importantly, specifically binding or accumulating in the cells, and being reliably detected. However, only a few meets all of these harsh restrictions. In this work, we propose a well-known natural compound, AXT, as a biosensor for visualization of various lipidic structures inside cells using Raman imaging. AXT is a xanthophyll wieldy spread in nature, responsible for the red colour of e.g. sea food, thus, it can be obtained from natural resources keeping the rules of green chemistry and its production is cheap and environmentally friendly.

AXT molecule contains long hydrocarbon chain responsible for its hydrophobic properties and high solubility in lipids. Lipids are omnipresent in cells and tissues but can be formed additionally upon e.g. cell inflammation. Due to the high scattering cross section of lipids, they can be detected based on their characteristic Raman profile, however, in some structures i.e. nuclear membrane, because of the diffraction limits and low resolution of Raman images, they cannot be visualized directly. The application of AXT helped to solve this issue. We showed that AXT accumulates in lipidic structures of HMEC-1 in time-dependent manner. AXT was observed in LDs after 30 min stimulation and for longer incubation times AXT enables visualization of the ER and even nuclear envelope. The affinity of AXT to lipids was proven in the followed experiments by applying high laser power and visualizing of lipids. This finding revealed that AXT can be used as a selective biosensor for lipidic structures for Raman imaging studies. Moreover, the contrast of obtained Raman images is even better than images obtained by an integration the marker bands of lipids.

On the other side, we have proven that AXT may have anti-inflammatory properties (decreased ICAM-1 overexpression) compatible with a possible beneficial effects of this compound on endothelium, including an improvement of lipid profile on the glucose metabolism in patients with hypercholesterolemia [26] and the possible prevention of age-related inflammatory diseases [27]. The results indicate that AXT is not inert to endothelial cells and this fact needs to be taken for a consideration.

This work opens the new chapter for AXT as a Raman probe and highlights its great potential in future studies on cellular or nucleus membranes damage, especially when fast Raman imaging techniques as stimulated Raman spectroscopy (SRS) or coherent antistokes Raman spectroscopy (CARS) would be applied.
